# Rediscovery of *Cyperus flavescens* (Cyperaceae) on the northeast periphery of its range in Europe

**DOI:** 10.7717/peerj.9837

**Published:** 2020-09-14

**Authors:** Paweł Marciniuk, Jolanta Marciniuk, Andrzej Łysko, Łukasz Krajewski, Justyna Chudecka, Janina Skrzyczyńska, Agnieszka Anna Popiela

**Affiliations:** 1 Faculty of Exact and Natural Sciences, Siedlce University of Natural Sciences and Humanities, Siedlce, Poland; 2 Faculty of Computer Science and Information Technology, Western Pomeranian University of Technology in Szczecin, Szczecin, Poland; 3 Department of Nature Protection and Rural Landscape, Institute of Technology and Life Sciences, Falenty, Poland; 4 Faculty of Environmental Management and Agriculture, Western Pomeranian University of Technology in Szczecin, Szczecin, Poland; 5 Faculty of Agrobioengineering and Animal Husbandry, Siedlce University of Natural Sciences and Humanities, Siedlce, Poland; 6 Institute of Biology, University of Szczecin, Szczecin, Poland

**Keywords:** *Cyperetum flavescentis*, Endangered species, Habitat, Ecology, Bioinformatics, Central Europe, Poland

## Abstract

In recent years, three large populations of *Cyperus flavescens* were found in Poland, the richest occurrence of this species in over 30 years. The goal of this research is to determine the habitat factors lead to the mass occurrence of *C. flavescens* and the present situation of that species and its habitat in Central Europe. Soil conditions of the three populations were studied. To determine the correlation between the occurrence and abundance of species and the chemical parameters of the soil, the DCA and CCA methods were used. The DCA of environmental Ellenberg values was made for all relevés known from Poland. The occurrence of *C. flavescens* in plant communities in Central Europe was studied. The maximum entropy method was used for potential distribution analysis of *C. flavescens*. All analyzed traits are important for this species and none has an advantage over another, so the environmental factor affecting the occurrence of *C. flavescens* is different from the tested. Analysis on Ellenberg values indicate that the longest gradients are temperature, moisture and nutrients. The analysis of vegetation data involving *Cyperus flavescens* available from Central Europe indicates that this species occurs mainly in the company of *Juncus bufonius* and* Plantago intermedia*, whereas other species of the Isoëto-Nanojuncetea class appear rarely. In MaxEnt analysis based on bioclimatic variables, the most important variable is BIO1 (Annual Mean Temperature). The results of our observation indicate that anthropogenic factors such as grazing livestock have a positive effect on the occurrence of the species. It is also very likely that the species is promoted by very warm summers with only short periods of heavy rains. A map of the potential distribution of *C. flavescens* in Central Europe created according historical and future data show an extension of the range of potential habitats to the north and east.

## Introduction

*Cyperus flavescens* L. (= *Pycreus flavescens* (L.) P.Beauv. ex Rchb.) is a species with a broad cosmopolitan range (Meusel, Jäger & Weinert, 1965; Jiménez-Mejıas, Luceño & Peterson, 2011). It is listed as an endangered taxon in many places in Europe, and is extinct in some regions (Bilz et al., 2011; Lansdown, 2013). In Europe, habitats of *C. flavescens* are widespread, especially in the south of Europe, in places with a high concentration of nitrogen compounds and mineral salts that are usually classified to the Isoëto-Nanojuncetea class (Ter Braak & Smilauer, 2002). *C. flavescens* usually grows on the edges of lakes and periodically flooded places, where the water level is variable and probably suppresses competition. It also occurs on wet and marshy meadows, on the banks of rivers, waterholes and on old rice fields (e.g., *Moor, 1936; Pietsch, 1973; Brullo & Minissale, 1998*).

Central Europe represents the northeastern periphery of this taxon’s actual range in Europe (*Hultén & Fries, 1986*). It is mentioned on red lists of Central European countries: it is critically endangered in the Czech Republic (*Grulich, 2012*), strongly endangered in Germany (*Ludwig & Schnittler, 1996*), and vulnerable in Slovakia (*Maglocký & Feráková, 1993*). The northernmost occurrences of *Cyperus flavescens* are in Poland, where it is classified as a species vulnerable to extinction (*Popiela, 2005; Popiela, Łysko & Konopska, 2014*), similar to its status in Germany and the Czech Republic (*Bettinger et al., 2013; Dřevojan & Šumberová, 2016*). In Poland, the species is associated with natural habitat 3130, namely flooded muddy river banks, and on sandy, periodically flooded, often pastured and trampled banks of rivers, oxbow lakes and lakes. Phytocenoses with *C. flavescens* are rarely recorded in Central Europe and this habitat is treated as disappearing (*Pietsch, 1973; Pietsch & Müller-Stoll, 1974; Popiela, 1997; Šumberová & Hrivnák, 2013*).

In recent years, three large populations of *Cyperus flavescens* were found by the authors of this research (J.M. P.M., Ł.K.). They are the richest species occurrences in over 30 years. Thus, the aim of this research is to determine (1) the habitat factors that determine the mass occurrence of *C. flavescens*, and (2) the present situation of this endangered species and its habitat in Central Europe measured against its potential range.

## Methods

### Potential distribution

The maximum entropy method was used Hernandez et al. (2006); Phillips, Anderson & Schapire (2006); Guisan et al. (2007); Wisz et al. (2008); Elith & Graham (2009) for potential worldwide distribution analysis of *Cyperus flavescens*. The range of the species was analyzed on the basis of all data in databases: Global Biodiversity Information Facility GBIF (2020) and from Popiela (1996) and Popiela, Łysko & Konopska (2014). Popiela, Łysko & Konopska (2014) in Poland. In total, 9086 geolocated worldwide occurrences were analyzed (8849 from GBiF and 237 from Poland).

The analysis was performed on climate data at a resolution of 2.5 (2.5 geographical minutes per single pixel). The occurrences of *C. flavescens* are grouped in such a way that there is only one occurrence in one pixel. In this way 3,099 pixels were formed. The PostgreSQL database ver. 12, using vector point geometry was used, which was then transformed into a regular 2,5 grid using the SAGAGIS ver. 6.3.0 software. The visualization of the results was performed in the QGIS 3.11 software. Statistical analyzes were carried out in MaxEnt ver. 3.4.1.

The jackknife MaxEnt analysis was used to indicate the most informative variables. The accuracy and performance of species distribution models were evaluated using threshold-independent receiver operation characteristic (ROC) analysis and the threshold dependent binomial test of omission (*Phillips, Anderson & Schapire, 2006; Elith et al., 2006*). The analyses were performed in 100 replicates for the MaxEnt model and 100 replication for the jackknife analysis. Testing was performed on 25% random points in relation to all sites of the *Cyperus flavescens*. The results are presented on an area under the ROC curve (AUC) ranging between 0 and 1. Models with an AUC value greater than 0.75 were considered acceptable models (*Pearce & Ferrier, 2000*). Omission rates in optimal models are less than 0.05 (*Anderson, Lew & Peterson, 2003*).

Analysis of the potential distribution of the species in current and future climatic conditions was carried out using the WorldClim data model (period 1970–2000) at a resolution of 5 × 5 km (Fick & Hijmans, 2017). The analysis of potential changes in distribution as a result of climate change was made on the basis of the CM6-SSP3-7.0-bc (https://worldclim.org/data/cmip6/cmip6_clim2.5m.html- model (period 2021 to 2040), which lies exactly in the middle of the range of baseline outcomes produced by energy system models. It assumes a gradual increase in warming due to CO_2_ emissions until 2040. The species is widespread in both temperate and tropical regions and since there is a huge discerepacy in the resolution of the data for different regions the potential dispersal is underestimated for tropical regions. Since the regional focus of this study, only the results for Europe are shown.

### Ecological research

**Figure 1:**
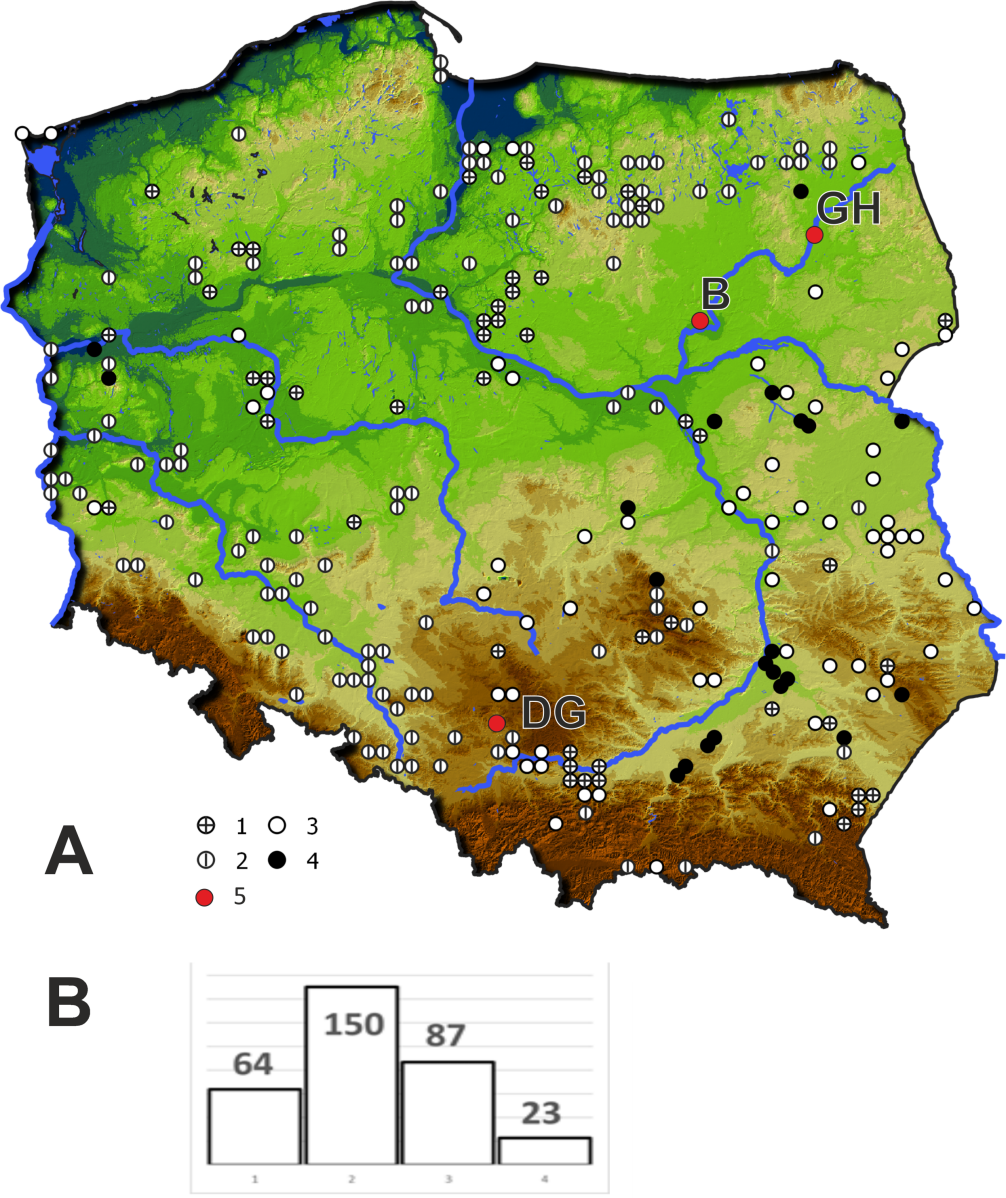
Location of the populations of *Cyperus flavescens* studied on the background of the distribution of the species in Poland (A), number of occurrences of *C. flavescens* in Poland in years 1847–2018 (*n* = 324). 1 –station before 1900, 2 –station in years 1900–1945, 3 –station in years 1946 –1989, 4 - station after 1990, 5 –populations studied: B - Bindużka, GH –Grobla Honczarowska, DG –Dąbrowa Górnicza.

In 2019 three populations of *Cyperus flavescens* were studied (Fig. 1):

(1) B^1^ 1* acronym for the population used in the text- located in the village of Bindużka (21.3350 long., 52.7921 lat..) on the extensive floodplain terrace of the Narew River (NE Poland), partly occupied by a large oxbow lake connected to the river. The area is used as an extensive pasture for cows. The population of *C. flavescens* occupies an area of 300 m^2^. The estimated population size is more than 10,000 individuals. *C. flavescens* occurs in a zone, strongly disturbed by grazing and periodic breakdown due to overdriving by community of the *Cyperetum flavescentis*.

(2) GH^1^ - Grobla Honczarowska (22.5550 long., 53.3055 lat.), in the Biebrza River marsh, Biebrzański National Park (NE Poland), many thousands of *C. flavescens* individuals occupy 7 km along a heavily used road;

(3) DG^1^ - Dąbrowa Górnicza (S Poland, (19.2370 long., 50.3606 lat.): (a) Kuźnica Warężyńska sand pit, in the eastern part, within the boundaries of PLH240037 “Lipienniki w Dąbrowie Górniczej”, there are many thousands of individuals on sandy roads, roadsides and other periodically flooded sites, populations also occur on the mineral shores of an artificial lake, (b) a beach on the northeast shore of the lake Pogoria I - in a sandpit that was flooded around 1943. The site was proposed for protection as a 3130 habitat in the Natura 2000 site “Lipienniki w Dąbrowie Górniczej”; total area (a) and (b) around 3,400 m^2^ with tens of thousands of *Cyperus flavescens* specimens in patches of *Cyperetum flavescentis*.

In each population, eight 1-m^2^ research plots were chosen, as far apart as possible. The plots were selected in such a way that: two plots were 60%–100% covered with *Cyperus flavescens*, two plots where the species covered 20%–60%, two plots where the *C. flavescens* coverage was below 20%, and two plots without *C. flavescens*. In each plot a relevé was made. In addition, with a soil stick, five probes were collected in each plot, then combined in a bag as a collective sample.

The soil samples were air-dried, disaggregated and sieved through a 2-mm mesh to separate skeleton and earth mass. In the earth mass of the samples (without skeleton) the following properties were analyzed by methods commonly used in soil science: texture (particle size composition) was determined using the aerometric method of Casagrande in the Prószyński modification; the textural class names were determined using the classification of the USDA (United States Department of Agriculture); pH in KCl was determined by the potentiometric method; electric conductivity (EC) of a soil suspension with a 1:2.5 soil/water ratio was determined by conductometric method; the content of organic carbon (C org) was determined by the Tiurin method; the content of total nitrogen (C tot) was determined by the Kjeldahl method; the content of available forms of phosphorus and potassium (P_2_O_5_, K _2_O) was determined by the Egner–Riehm method; the content of available forms of magnesium (Mg) was determined by the Schachtschabel method.

To determine the correlation between the occurrence and abundance of species and the chemical parameters of the soil, Juice v.7.0.213 software and the Detrended Correspondence Analysis (DCA) and Canonical Correspondence Analysis (CCA) methods created by Canoco 4.52. were used. Initially DCA analyses were performed. Because of the small dataset, a selection method by segments without transformation method was used. *Cyperus flavescens* received a higher weight in the analysis (2.0), and other species received a 1.0.

The unimodal CCA method with inter-species distance and biplot scaling (Lá) was used to check the relationship between the occurrence of species and environmental variables. Because of the small dataset, species were not transformed, and *C. flavescens* had a high weight applied in the analysis (2.0 whereas other species had a weight of 1.0). To indicate the weight of individual environment variables, Monte Carlo permutation tests (*n* = 499) with restricted spatial structure were used.

Moreover, the Detrended Correspondence Analysis (DCA) of environmental Ellenberg values was made for all relevés published in Poland (45 relevés) that were performed in the years 1961–2018 (*Śliwińska, 1961; Faliński, 1966; Fijałkowski & Kozak, 1970; Hereźniak, 1972; Fijałkowski, 1978; Ochyra, 1985; Cabała, Paul & Piątek, 2004; Nobis & Michalewska, 2004)*, and personal observations of the authors (J Marciniuk, P Marciniuk 2018–2019., Ł Krajewski, 2018–2019; see Table 4) using Juice 7.0 software. In addition, 213 and R statistical environment with the Vegan package were used. For interpretation of the main environmental gradients, average non-weighted Ellenberg indicator values for light, temperature, continentality, soil reaction, moisture and nutrients (*Ellenberg et al., 1992*) were used as supplementary variables.

### Synoptic table

The occurrence of *Cyperus flavescens* in plant communities in Central Europe (Poland, southeastern Germany, the Czech Republic and Slovakia) was studied using all available data. The data from the Czech Republic and Slovakia were obtained from the Slovak Vegetation Database (*Šibík, 2012*) and the Czech National Phytosociological Database (*Chytrý & Rafajová, 2003*). The published dataset was supplemented with the authors’ personal observations (J Marciniuk, P Marciniuk 2018–2019., Ł Krajewski, 2018–2019; see Table 4). Ninety-four relevés were used. Species with a constancy > 20%, are shown, except for the taxa of the Isoëto-Nanojuncetea class, where all species are included.

The nomenclature of Mirek et al. (2002) Ochyra, Żarnowiec & Bednarek-Ochyra (2003), Szweykowski (2006), and Mucina & Tichý (2016) was followed. Herbarium materials were deposited in SZUB and WSRP.

Field work was carried out with the following permits:

Biebrza National Park (permission no. 51/0/2019) 211

the Ministry of the Environment (permission no. R/2102/2019)

the Regional Directorate for Environment Protection in Katowice (permission no. 6400.6.2019.MS.1) 212. During this research, equipment from the Center of Molecular Biology and Biotechnology of the University of Szczecin was used.

## Results

In jackknife MaxEnt analysis based on bioclimatic variables to the year 2017, the environmental variable with highest gain when used in isolation is BIO1 (Annual Mean Temperature), which therefore appears to have the most useful information by itself.

**Figure 2:**
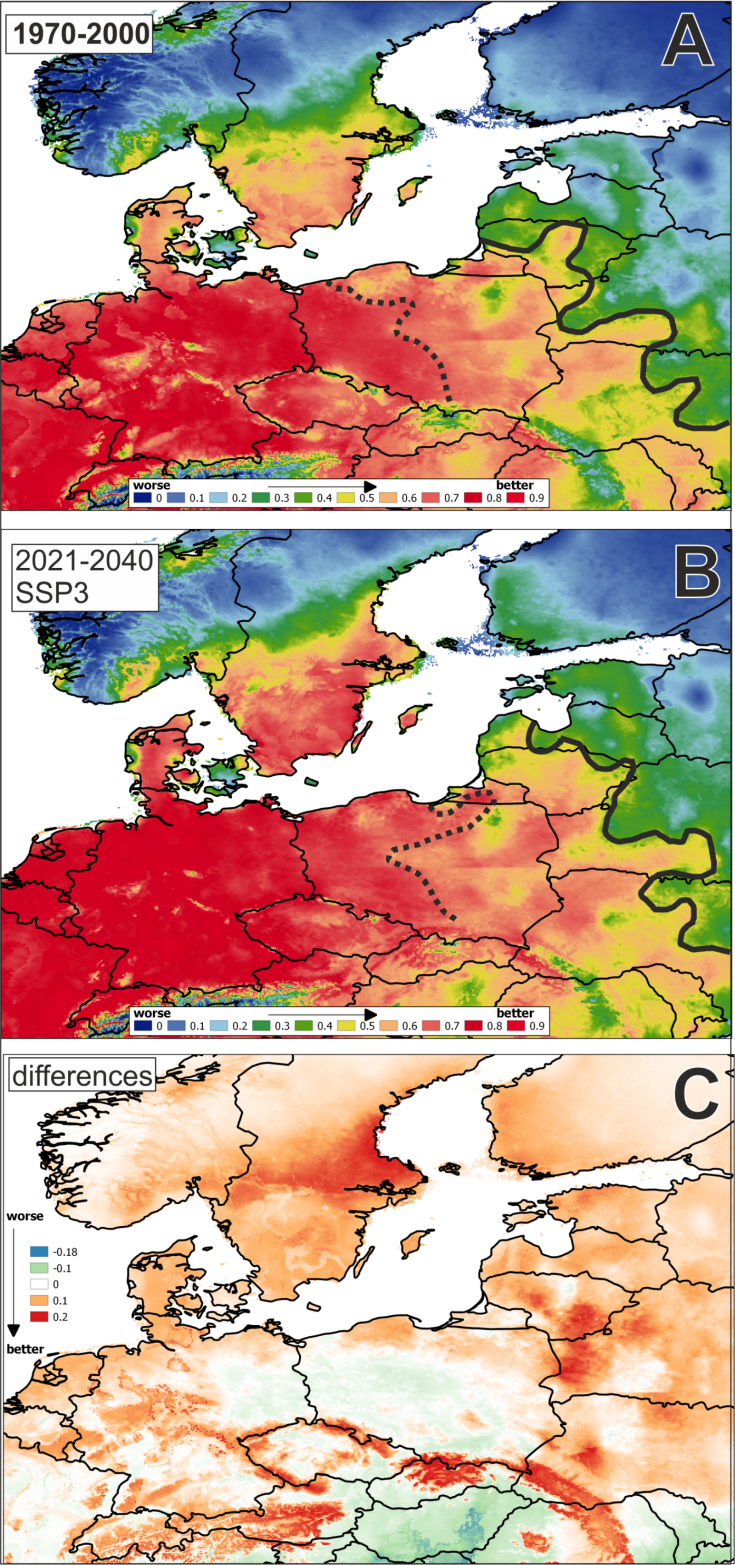
Potential distribution area of *Cyperus flavescens* (mean AUC: in years: (A): 1970–2000 = 0.887; (B): 2021–2040 = 0.888, 1 = 100%). The black dashed line AUC < =0.75; black solid line AUC < = 0.5. Potential distribution in period: (A): 1970–2000, (B): 2021–2040; (C): difference: B-Adata used to perform the analyses: period 1970–2000 - WorldClim (Fick & Hijmans, 2017); period 2021–2040 - CMIP6 (wc2.1_2.5m_bioc_CNRM-CM6-1_ssp370_2021-2040).

The average training AUC for the replicate runs is 0.8868, and the average standard deviation is 0.0037 (Fig. 2).

The result of the analysis for future bioclimatic variables using models from the years 2021 to 2040 is similar. The environmental variable with highest gain when used in isolation is BIO1, which therefore appears to have the most useful information by itself (AUC 0.8875, average standard deviation is 0.0036).

A map of the potential distribution of *Cyperus flavescens* in Central Europe, created according historical and future data, shows an extension of the range of potential habitats to the north and east (Fig. 2).

**Figure 3:**
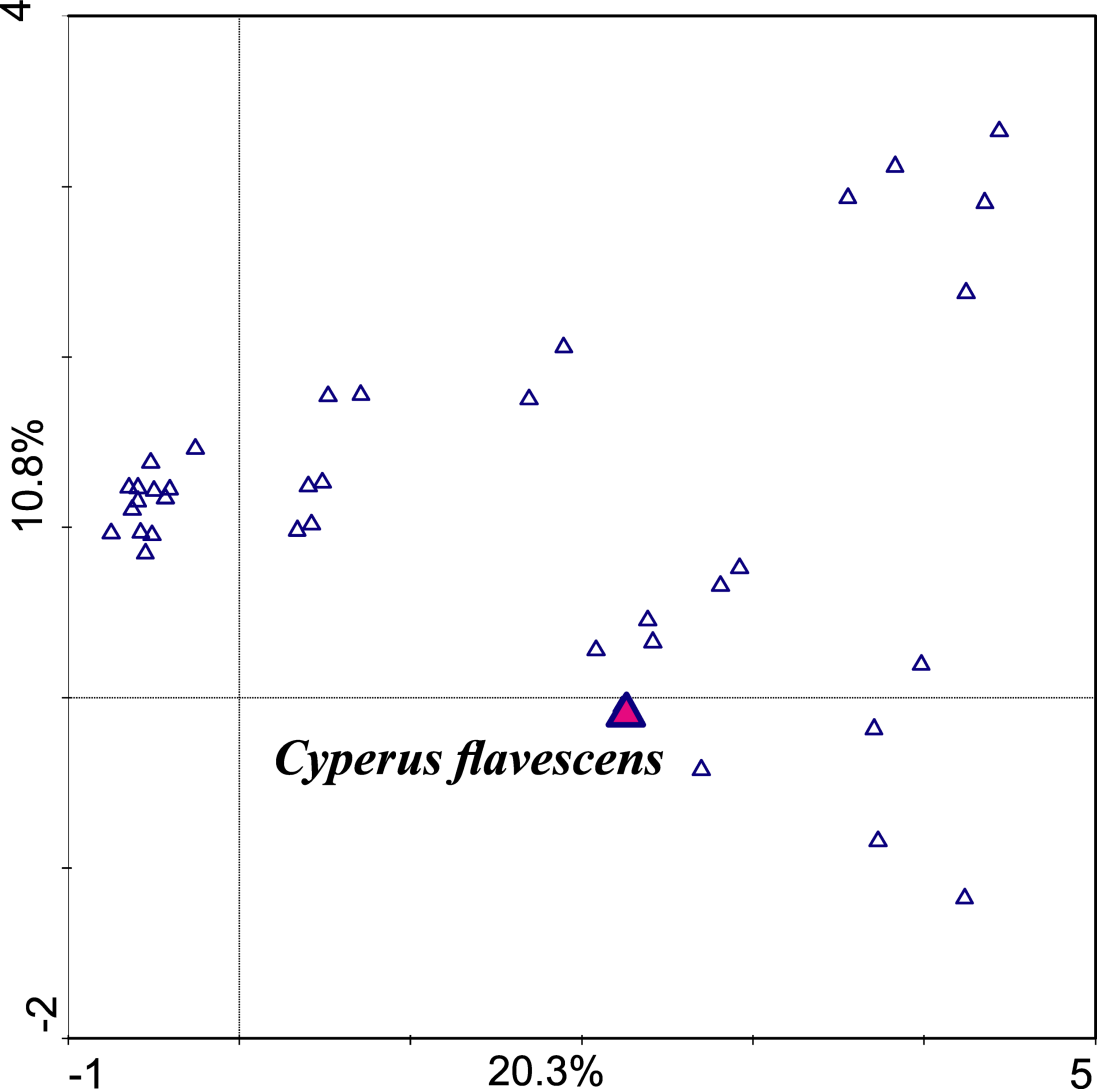
Ordination plot of DCA unimodal analysis checking correlation between species (first axis explains 20.3% variability, the second 10.8%).

An examination of the correlation between the occurrence and abundance of species and the chemical parameters of the soil showed that the gradient length represented by the first consulting axis is 3.506 SD. This means that along this gradient, the species does not decompose in the full spectrum of the Gauss curve. This result showed that the dataset can be analyzed using linear and unimodal techniques (Jongman, Ter Braak & Van Tongeren, 1987; Brullo & Minissale, 1998). The first axis is the most important; it takes into account the differences in sets and explains 20.3% of the variability; the second axis explains 10.8% (Table 1, Fig. 3).

**Table 1:** Results of DCA analysis checking relationships between species.

Axes	1	2	3	4	Tot. Interia
Eigenvalues	0.634	0.335	0.155	0.093	3.119
Lengths of gradient	3.506	2.046	1.608	1.413	
Cumulative percentage variance of species data	20.3	31.1	36.0	39.0	
%	20.3	10.8	4.9	3.0	

**Notes.**

* SD to Cyperus flavescens = 3.2428.

The analyses made shown that the substrate for *Cyperus flavescens* growth and development on the three examined locations was loose sand (sand acc. USDA). The contents of the granulometric fractions were: 91%–98% for the sand fraction, 1%–8% for the silt fraction, and 0%–2% for the clay fraction. Sands are very dry substrates; they are periodically wet if they are located in terrain with depressions, valleys, or floodplain areas. Soil pH values most often indicated an alkaline character, and less often, a neutral or slightly acidic character (Table 2). The EC values were quite variable within the locations; however, they indicated a low level of soil salinity. The soils were very poor in available potassium, and often poor in available phosphorus. However, the available magnesium content was often very high and high.

**Table 2:** Results of the Monte Carlo analysis used with CCA unimodal method.

Variable	Lambda	LambdaA	P	F
	Marginal effects	conditional effects		
pH	0.42	0.42	0.014^*^	3.46
P_2_O_5_ [mg 100 g^−1^of soil]	0.37	0.26	0.050^*^	2.19
EC [µS cm^−1^]	0.34	0.17	0.080	1.54
Mg [mg 100 g^−1^of soil]	0.25	0.14	0.208	1.22
K_2_O [mg 100 g^−1^of soil]	0.22	0.12	0.460	1.07
N tot [%]	0.22	0.09	0.522	0.79
C org [%]	0.21	0.07	0.804	0.65
C/N	0.12	0.06	1.000	0.46

**Notes.**

* *P* ≤ 0.05.

Canonical correspondence analysis (CCA) as well as the Monte Carlo permutation show that the Ec and C org variables had the most correlation. Therefore, Ec was deleted from further analysis. Only pH and P_2_O_5_ were statistically significant, which explains the variability of the species in individual phytosociological images (*p* ≤ 0.05; pH = 13.46% variation; P_2_O_5_ = 8.33%) (Table 2).

**Figure 4:**
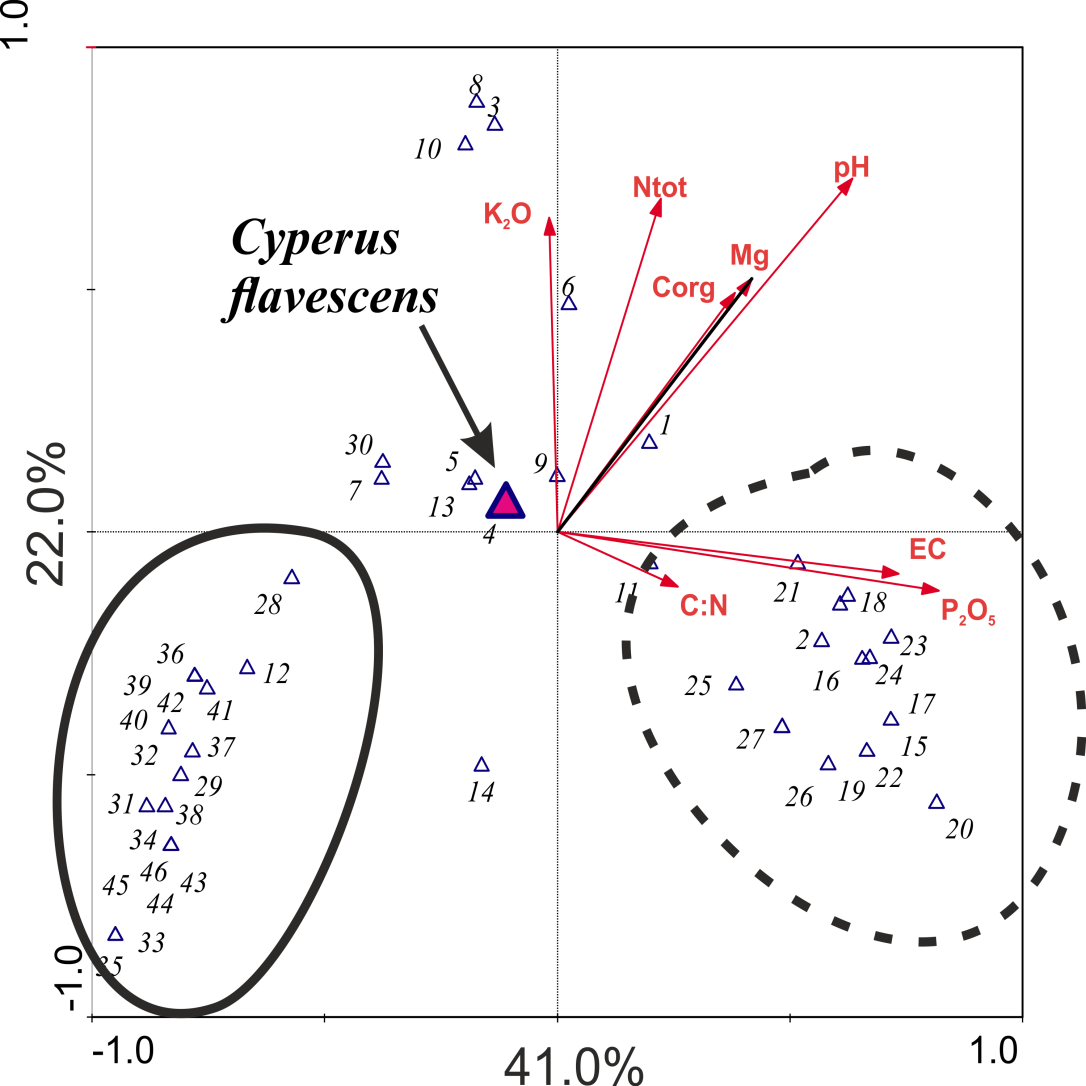
Ordination plot the CCA analysis checking the relationship between species and soil chemical parameters. 1-*Agrostis stolonifera*; 2-*Bidens tripartita*; 3-*Carex hirta*; 4-***Cyperus flavescens***; 5-*Juncus articulatus*; 6-J*. bufonius*; 7-*J. tenuis*; 8- *Plantago major*; 9- *P. intermedia*; 10- *Polygonum aviculare*; 11- *Trifolium repens*; 12-*Phragmites australis*; 13- *Poa annua*; 14- *Bidens frondosa*; 15- *Cyperus fuscus*; 16- *Echinochloa crus-galli*; 17-*Polygonum lapathifolium*; 18-*Sagina procumbens*; 19-*Bidens cernua*; 20-*Mentha arvensis*; 21-*Veronica scutellata*; 22-*Potentilla anserina*; 23-*Gnaphalium uliginosum*; 24-*Myosotis palustris* agg.; 25-*Alopecurus geniculatus*; 26-*Eleocharis acicularis*; 27-*Rorippa palustris*; 28-*Isolepis setacea*; 29-*Carex viridula*; 30-*Phalaris arundinacea*; 31-*Hypochoeris radicata*; 32-*Leontodon autumnalis*; 33-*Alisma plantago-aquatica*; 34-*Lycopus europaeus*; 35-*Lysimachia vulgaris*; 36-*Centaurium erythraea*; 37-*Centunculus minimus*; 38-*Cirsium palustre*; 39-*Drosera rotundifolia*; 40-*Eupatorium cannabinum*; 41-*Lythrum salicaria*; 42-*Schoenoplectus tabernaemontani*; 43-*Holcus lanatus*; 44-*Juncus alpino-articulatis*; 45-*Plantago lanceolata*; 46-*Salix repens* ssp*. rosmarinifolia*.

The result of the CCA analysis, which evaluates the relationship between species and soil chemical parameters, indicates that the most important variables for the analyzed dataset are pH and P_2_O_5_, which indicate strong alkalization of the habitat. However, the P_2_O_5_ and EC associated with the first consulting axis are responsible for highest species variability in the habitats in total; this axis explains 41% of species diversity in the study areas. The second consulting axis is most strongly associated with pH and with K_2_O and Ntot. This axis accounts for 22% of variation (Table 3). *Cyperus flavescens* is placed very close to the center of the axis, which indicates that all analyzed traits are important for this species and none has an advantage over another. However, two groups of species were distinguished: one was strongly associated with variable pH and the other was associated with P_2_O_5_ content (Fig. 4).

**Table 3:** Results the unimodal CCA analysis: correlation of species and environmental variables.

Axes	1	2	3	4	Tot. Interia
Eigenvalues	0.545	0.292	0.152	0.103	3.119
Species-environment correlations	0.953	0.919	0.765	0.881	
Cumulative percentage variance: - of species data	17.5	26.8	31.7	35.0	
- of species-environment relations	41.0	63.0	74.5	82.2	
Sum of all eigenvalues	3.119				
Sum of all canonical eigenvalues	1.327				

**Figure 5:**
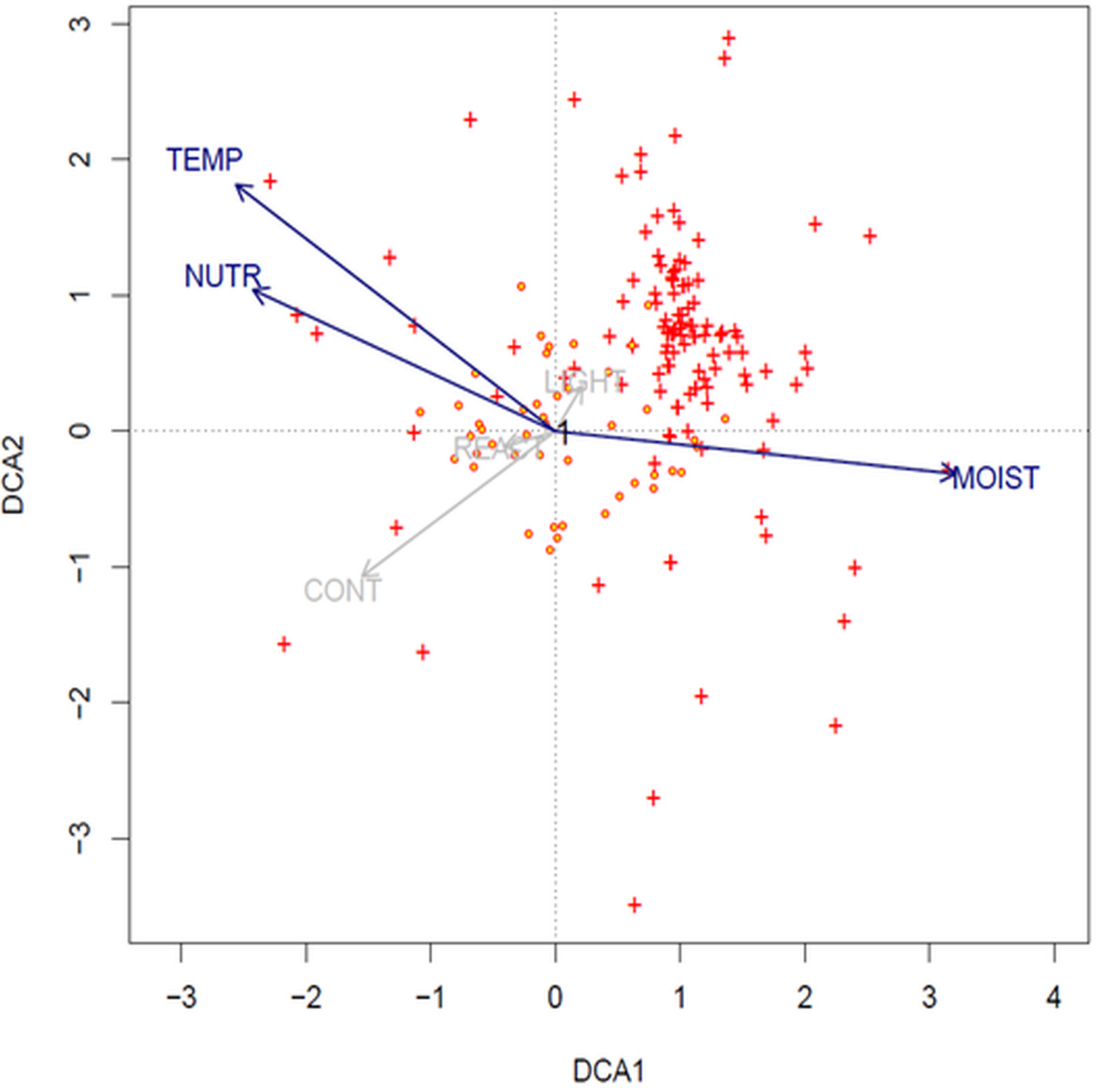
DCA ordination biplot (1st and 2nd axis) with projected relevés (red-yellow circles) and species (red crosses) (options: downweighting of rare species and transformation log(*Xi*, *j* + 1)). Ellenberg indicator values: TEMP, temperature; REACT, soil reaction; LIGHT, light; CONT, continentality; NUTR, nutrients; and MOIST, moisture.

The result of DCA analysis on Ellenberg values indicates that the longest gradients are temperature, moisture and nutrients (SD of the axis: DCA1: 4.5; DCA2: 4.2). Values of the species are clustered and concentrated mostly with the first ordination axis (moisture), and with the second axes (temperature and nutrients) (Fig. 5).

**Table 4:** Synoptic table of the vegetation groups (1–7) characterized by the presence of *Cyperus flavescens* (the table contains species reaching more than 20% of the presence only, with an exception of species if the Isoëto-Nanojuncetea class). 1 Ł. (Krajewski, 2016; Krawczyk et al., 2016) unpubl., 2 –J. Marciniuk, P. Marciniuk 2018, unpubl., 3 –(Krajewski, 2016), 4 –(Pietsch & Müller-Stoll, 1974), 5 –(Śliwińska, 1961; Fijałkowski & Kozak, 1970; Hereźniak, 1972; Fijałkowski, 1978); Ochyra, 1985, Cabała, Paul & Piątek, 2004; Nobis & Michalewska, 2004) ; 6–Slovak Vegetation Database (Šibík, 2012), 7–Czech National Phytosociological database (Chytrý & Rafajová, 2003).

	1	2	3	4	5	6	7
	PL	PL	PL	GER	PL	SLO	CzR
Numer of relevés	6	10	11	8	17	23	19
Ch. Ass. Cyperetum flavescentis							
*Cyperus flavescens*	V	V	V	IV	V	V	V
*Carex viridula*	I		IV		II		II
*Sagina nodosa*			I		III		
*Centaurium pulchellum*				III		I	
**Ch. All. Eleocharition soloniensis**							
*Cyperus fuscus*	I	I	I	IV	II	I	II
*Peplis portula*				III	II	I	III
*Carex bohemica*				II	I		II
*Eleocharis ovata*					I		III
*Eleocharis acicularis*			I			II	II
*Limosella aquatica*					I	I	I
Sporadic: *Dichostylis micheliana* 6 (I), *Elatine hydropiper* 7 (I), *Lindernia procumbens* 7(II)							
Ch. All. Radiolion linoidis							
*Centunculus minimus*			II		I	III	
*Gypsophila muralis*				II			I
*Radiola linoides*			I			II	
*Hypericum humifusum*				II			
Sporadic: *Anthoceros punctatus* 5(I), *Phaeoceros laevis* 5(I)							
Ch. O. Nanocyperetalia, Cl. Isoëto-Nanojucetea							
*Juncus bufonius*	V	V	II	V	III	I	III
*Plantago intermedia*	V	V	V	V	II	I	III
*Gnaphalium luteo-album*				II		I	I
*Gnaphalium uliginosum*		I		V	III	II	II
*Isolepis setacea*	I		II		I	II	I
*Potentilla supina*						I	I
*Juncus bulbosus*			I			I	I
Sporadic: *Centaurium erythraea* 2(I), *Crassula aquatica* 7(I), *Juncus capitatus* 6(I), *J. tenageia* 6(I), *Lindernia dubia* 7(II), *Lythrum hyssopifolia* 6(II), *Mentha pulegium* 6(I), *Physcomitrella patens* 4(II), *Physcomitrium pyriforme* 4(II), *Tortula truncata* 4(II),							
Ch. Cl. Molinio-Arrhenatheretea							
*Juncus articulatus*	V	III	V	II	III	II	III
*Agrostis stolonifera*	V	V	II	V	II	III	I
*Trifolium repens*	I	III		V	II	I	II
*Poa annua*	I	III		IV	II	I	
*Juncus tenuis*	I		II	IV	I		
*Odontites serotina*		II		IV			
*Molinia caerulea*			I	II	I		
*Juncus compressus*			II	IV			
*Carex hirta*	I	III			I	I	
*Lythrum salicaria*	I		II		I	I	I
*Mentha arvensis*		III			I	I	
*Plantago lanceolata*			II		I		
*Prunella vulgaris*				IV	I	I	
*Ranunculus repens*		I		III		I	
*Sagina procumbens*				III	I	II	III
*Lotus corniculatus*				III		II	I
*Briza media*					I	I	
*Mentha aquatica*				II		II	
*Carex panicea*				II		I	I
*Trifolium pratense*	I				I	I	
*Potentilla anserina*		III		V	II	II	I
*Agrostis canina*					II		I
*Ranunculus flammula*					III		II
*Linum catharticum*	III				I		
*Alopecurus geniculatus*				IV		I	
*Plantago major*	I			V	I	I	
*Lolium perenne*				IV	I		
*Achillea millefolium*					I	I	
*Cerastium holosteoides*					I	I	
*Holcus lanatus*					I	II	I
*Leontodon autumnalis*					I		I
*Ranunculus acris*					I	I	
*Festuca pratensis*					I	I	
*Lychnis flos-cuculi*					I	I	
Spradic: *Agrostis gigantea* (II), *Blysmus compressus* 4(II), *Equisetum palustre* 1 (IV*), Lysimachia nummularia* 6(II), *Polygonum aviculare* 4(IV) *Potentilla reptans* 6(I), *Rumex acetosella* 5(II), *R. crispus* 6(II), *Trifolium fragiferum* 4(IV),							
Ch. Cl. Bidentetea							
*Bidens tripartita*	II	IV			I	III	II
*Bidens cernua*		II			II	I	I
*Polygonum persicaria*		IV			I	II	
*Chenopodium glaucum*						I	I
*Polygonum hydropiper*					II	II	III
*Polygonum lapathifolium*	I					I	III
*Chenopodium rubrum*						I	I
*Ranunculus sceleratus*						I	I
*Rorippa palustris*					I		II
*Alopecurus aequalis*						I	I
Sporadic: *Bidens frondosa*: 3(IV)							
Ch. Cl. Phragmito-Magnocaricetea							
*Eleocharis palustris*	I	I	II		I		
*Phragmites australis*	II		IV		II		
*Alisma plantago-aquatica*			I		I	I	I
*Glyceria fluitans*		I		II		I	
*Lycopus europaeus*	I		IV		I	II	II
*Veronica anagallis-aquatica*					I	II	I
Sporadic: *Bolboschoenus maritimus* 6(III), *Leersia oryzoides* 7(III), *Poa palustris* 6(II)							
Ch. Cl. Scheuchzerio palustris-Caricetea fuscae							
*Equisetum variegatum*			I		I	I	
*Eleocharis quinqueflora*	I			II	I		
*Triglochin palustris*	I			II	I		I
*Hydrocotyle vulgaris*				II			
*Juncus alpinus*	I		II				
*Alisma lanceolata*						I	
*Veronica scutellata*					I		I
*Pinguicula vulgaris*				II			
Ch.Cl. Juncetea maritimi							
*Carex scandinavica (C. serotina subsp. pulchella)*					V		
*Carex distans*				II			
Ch. Cl. Papaveretea rhoeadis							
*Echinochloa crus-galli*		II	I	II	I	I	IV
*Anthemis arvensis*						I	I
*Chenopodium album*						I	
*Conyza canadensis*					I	I	I
Other							
*Chenopodium ficifolium*						I	I
*Calliergonella cuspidata*	I				II	I	
*Salix cinerea*					I		I
*Sparganium emersum*					I		I
*Potentilla erecta*					I	I	
*Pohlia* sp*.*	II						I
*Riccia cavernosa*	I						I

**Notes.**

Sporadic: *Bryum pseudotriquetrum* 1 (II), *Carex stenophylla* (II), *Dreplanocladus polycarpos* 1(II), *Drosera rotundifolia* 3(II), *Eupatorium cannabinum* 1 (II), *Hieracium pilosella* 6(II), *Plantago media* 6(II), *Riccia glauca* 4(II), *Salix repens* s.l. 3(III).

The analysis of the vegetation data available from Central Europe and involving *Cyperus flavescens* (Table 4) indicate that this species occurs mainly in the company of *Juncus bufonius* and *Plantago intermedia.* Other species of the Isoëto-Nanojuncetea class rarely appear. The list of species is complemented by taxa associated with the classes Molinio-Arrhenatheretea, Bidentetea, Phragmito-Magnocaricetea and Scheuchzerio palustris-Caricetea fuscae, where the highest constancy is achieved by *Juncus articulatus* and *Agrostis stolonifera.*

## Discussion

The results of the potential coverage modeling analysis of *Cyperus flavescens* show that Central Europe is at its northern end of potential occurrence (average AUC, > 0.75). It is assumed that species located on the edge of their area have a narrower ecological amplitude than the central populations (*Brown, 1984*). This could be a result of a “relative constant habitat rule” (*Walter & Walter, 1953*), i.e., a change of habitat as a compensation of climatic changes and maintenance of possibly constant environmental conditions. That change, or because of a lower genetic variability of the border populations, leads to a narrowing of the ecological amplitude and of accessible ecological niches (Van Valen, 1965). However, the results of some research enable the formulation of a different hypothesis: taxa with a lager distribution have broader ecological amplitudes on the northern borders of their geographical areas, which seems to be associated with reduced competition (*Diekmann & Lawesson, 1999*). In addition, higher extinction risks of species at the edge of their range are often mentioned (e.g., Bahn et al., 2006). Over the last 100 years, for reasons that are unclear, *C. flavescens* has lost about 90% of its locations in Central Europe (*Bettinger et al., 2013; Popiela, Łysko & Konopska, 2014; Dřevojan & Šumberová, 2016*). From descriptions in the literature and information on herbarium specimens’ labels (*Popiela, 1996*), it appears that in Poland, mainly sites on the lake shores disappeared, because of what could be linked with the progressive eutrophication of waters during the 20th century (Fig. 1). Moreover, nowadays, cattle do not regularly graze near the lakes, which results in a lack of trampling, initial places, deprived of rushes reaching the water. Such local extinction could mean the beginning of the disappearance of the species throughout Europe past of its range. Another problem is the difficulties in the field research: the taxon is very small and annual, hard to find, and its growth areas fluctuate (it appears in different locations); hence, it can be easily overlooked by researchers. For these reasons, *C. flavescens* is included in the IUCN red list of endangered species in Europe with the category of Lc (the lowest risk) because of its wide range and lack of identified global threats (*Lansdown, 2013*). However, extinction of this species has been observed in some European countries, especially where it has always been rare (*Hodálová, Feráková & Procházka, 1999; Lansdown, 2013; Nikolić & Topić, 2005; Popiela, Łysko & Konopska, 2014; Kaźmierczakowa et al., 2016*).

The number of *Cyperus flavescens* ‘ individuals within populations existing in Poland was always low (from a few to 300), and the species occupied small areas (*Popiela, Łysko & Konopska, 2014; Krawczyk et al., 2016*). In this context, the currently studied populations (B, HG, DG) might be the largest in the northeast periphery of the potential range of the taxon; hence, the current research is important for a better understanding of the ecological requirements and preservation of this species.

In the light of this research, none of the examined soil factors is decisive for the mass occurrence of *Cyperus flavescens*. The species is placed very close to the center of the axis, which indicates that all analyzed traits are important for this species and none has an advantage over another (Fig. 4). However, temperature, moisture and nutrients are important factors for the environment in which it occurs (Fig. 5). This may mean that the environmental factor affecting the occurrence of *C. flavescens* is different from the tested factors. It may turn out that anthropogenic factors, as well as soil properties (see the Results section) have a positive effect on the occurrence of the species. Population B occurs on roads periodically driven by vehicles and on the shores of a large oxbow of the Narew River, which are regularly edged and grazed by cattle. The habitat is flooded during the spring swells of the river and then remains dry during the summer and autumn. *C. flavescens* occurs in large numbers, achieving maximum density in quite dry and disturbed places. In GH, *C. flavescens* occurs abundantly, but the population develops before the mowing season of the neighboring sedge meadows (late summer); at the time of the mowing, the route is intensively used. Similarly, in the DG, the species is enhanced by anthropogenic disturbances: it occurs mainly in the sands on dirt roads that are periodically inundated after heavy rains and disturbed by vehicles, thus developing only a thin mud layer, at most. This environment allows for patches of pioneer vegetation. *C. flavescens* is also present on the sandy shore of an artificial water reservoir in an old sand quarry that is regularly used as a recreational beach (city swimming place); the taxon is most abundant here before and after the summer holidays (see also Krajewski, 2016).

The occurrence of the *Cyperus flavescens* seems to be strongly dependent on zoopressure, which maintains the initial nature of its habitat. It might be that under completely natural conditions, the occurrence of *C. flavescens* is limited to the rather heavily pitched waterways used by large herbivorous animals; the positive effect of herbivores on the population of *C. flavescens* was observed also by Taylor et al. (1994). In B, the replacement for wild herbivores is cattle. In the whole Narew River bend, grazing animals have wide access to the oxbow lake and the river, which means that the pressure is probably too weak. Therefore, the population of *C. flavescens* is limited to the small portion of the habitat used as a periodic (only at very low water levels) path through the oxbow lake. In B, this additional pressure seems to be a necessary factor for the stability of the population. It seems, that zoochory/anthropochory is also an important factor of spreading. For example, in GH, *C. flavescens* occurs along several kilometers of road, but it was not observed in neighboring plant communities. Similarly, in DG, the taxon appears most often at sandy roadsides (*Krajewski, 2016*). The species benefits from accidental transport of seeds to new habitats along roads, and in optimal conditions, the seeds subsequently germinate.

Habitats with occurrences of *Cyperus flavescens* are widespread in the south of Europe. The species occurs in places with a high concentration of nitrogen compounds and mineral salts, as well as on relatively short flooded and fairly compacted habitats, including anthropogenic ones, e.g., roads. The taxon occurs in many associations with the Isoëto-Nanojuncetea class (e.g., Brullo & Minissale, 1998; Ninot et al., 2000; Niculescu, 2016). In the Central Europe, unlike in its central range in Europe (*see* Brullo & Minissale, 1998), *C. flavescens* is not present in various associations but creates its own community, strongly referring to the *Cyperetum flavescentis* (Koch, 1926) association. *Centaurium pulchellum* is often missing in communities developing in Central Europe. In addition, the participation of the Radiolion linoidis alliance and the Eleocharition soloniensis alliance character species is small (Table 4) compared with the patches in the class’s central range (*see Brullo & Minissale, 1998*); like the other syntaxa from Isoëto-Nanojuncetea, *Cyperetum flavescentis* appears here in an impoverished form (*Popiela, 2005*). Libbert (1932) described patches of *Carex viridula* and *Centaurium pulchellum* from Western Pomerania (NW Poland), which he considered a very poor form of *Cyperetum flavescentis*.

Until now *Cyperetum flavescentis* has been rarely recorded in Central Europe, and this habitat has been treated as disappearing; patches of growth usually developed on periodically flooded lakes with crushed and grazed vegetation, peat pits, and the edges of ponds and streams (*Pietsch, 1973; Pietsch & Müller-Stoll, 1974; Popiela, 1997*). Among the 94 relevés listed in the synoptic (Table 4), 30 relevés (31%) were taken in Poland in the years 2004–2018 at new locations, meaning that habitat 3,130 is renewing and Poland can be the center of its preservation in this part of the continent. It should be noted that, so far, *Cyperetum flavescentis* were documented in Poland with only 10 relevés taken between 1932 and 1985 (*Popiela, 1997; Popiela, 2005; Popiela, Łysko & Konopska, 2014*). These communities probably no longer exist. Analysis of the ecological characteristics of this association occurring on the periphery of the potential range of *Cyperus flavescens* is problematic because the number of relevés is still small; however, analysis of Ellenberg indicator values has shown clear clustering of species mostly concentrating in areas with high moisture, temperature and nutrient axes (Fig. 5).

## Conclusion

Our research did not allow us to definitively resolve the reason for the rebirth of *Cyperus flavescens* in Poland. None of the examined soil factors namely pH, P_2_O_5_, EC, Mg, K_2_O, N tot, C org, C/N) is decisive for the mass occurrence of *Cyperus flavescens*. It may be an effect of anthropogenic changes (narrowing of the ecological amplitude) on the periphery of its potential range of the habitat, as has already been seen in other species of the Isoëto-Nanojuncetea class, namely *Radiola linoides, Centunculus minimus,* and *Illecebrum verticillatum* (*Popiela, 2005*). It is possible the species is enhanced by very warm summers with only short periods of heavy rain, which are now observed in Poland more often than ever before. Especially regarding the results of the MaxEnt analysis, the most important variable is BIO1 (Annual Mean Temperature). Over the years, the species has lost many habitats throughout Poland, and it is now limited to a small area of the country. The taxon’s range could be re-established in favorable climatic conditions and with certain forms of anthropopressure in the agricultural landscape. The problem of C4 species promotion caused by global warming has been raised many times (e.g., Lattanzi, 2010). Likewise, results of the comparative analysis of MaxEnt analysis using historical data and a predicted future model CM6-SSP3-7.0-bc indicate that the range of *Cyperus flavescens* in Europe will increase by 2040 as a result of global warming, especially in the north and east.

##  Supplemental Information

10.7717/peerj.9837/supp-1 Supplemental Information 1 Calculated Ellenberg values for releves

10.7717/peerj.9837/supp-2 Supplemental Information 2 Releves for calculate Ellenberg values

10.7717/peerj.9837/supp-3 Supplemental Information 3 Geographical data for maxent analysis

10.7717/peerj.9837/supp-4 Supplemental Information 4 Raw data for correlation between soils and releves

## Additional Information and Declarations

### Field Study Permissions

The following information was supplied relating to field study approvals (i.e., approving body and any reference numbers): The Biebrza National Park (permission nr 51/0/2019), Ministry of the Enviroment (permission nr R/2102/2019), and the Regional Directorate for Environment Protection in Katowice (permission nr 6400.6.2019.MS.1) approved this research.

### Data Availability

The following information was supplied regarding data availability: Raw data is available in the Supplementary Files.
